# Relationship between serum calprotectin (S100A8/9) and clinical, laboratory and ultrasound parameters of disease activity in rheumatoid arthritis: A large cohort study

**DOI:** 10.1371/journal.pone.0183420

**Published:** 2017-08-23

**Authors:** Jana Hurnakova, Hana Hulejova, Jakub Zavada, Petra Hanova, Martin Komarc, Herman Mann, Martin Klein, Olga Sleglova, Marta Olejarova, Sarka Forejtova, Olga Ruzickova, Jiri Vencovsky, Karel Pavelka, Ladislav Senolt

**Affiliations:** 1 Institute of Rheumatology, Prague, Czech Republic; 2 Department of Rheumatology, 1st Faculty of Medicine, Charles University, Prague, Czech Republic; 3 Department of Methodology, Faculty of Physical Education and Sport, Charles University, Prague, Czech Republic; University of Texas at Austin, UNITED STATES

## Abstract

**Background:**

Calprotectin may be a sensitive biomarker of rheumatoid arthritis (RA) disease activity.

**Objectives:**

In the current study, we investigated whether calprotectin is a better biomarker than CRP for predicting clinical activity and ultrasound parameters in patients with RA.

**Methods:**

A total of 160 patients with RA underwent clinical (swollen joint count—SJC, tender joint count—TJC, Disease Activity Score—DAS28, Clinical Disease Activity Index—CDAI, and simplified Disease Activity Index—SDAI) and ultrasound (German US7) examination. Clinical and laboratory measures were correlated with ultrasound findings using Spearman´s correlation coefficient. Differences in serum calprotectin levels in patients with variable disease activity according to the DAS28-ESR and CDAI scores were assessed using ANOVA. Multivariate regression analysis was used to determine the predictive values of calprotectin, CRP and SJC for CDAI and PD US synovitis scores.

**Results:**

Serum calprotectin was significantly associated with DAS28-ESR (r = 0.321, p<0.001), DAS28-CRP (r = 0.346, p<0.001), SDAI (r = 0.305, p<0.001), CDAI (r = 0.279, p<0.001) scores and CRP levels (r = 0.556, p<0.001). Moreover, calprotectin was significantly correlated with GS (r = 0.379, p<0.001) and PD synovitis scores (r = 0.419, p<0.001). The multivariate regression analysis showed that calprotectin is a better predictor of the CDAI score and PD US synovitis than CRP.

**Conclusions:**

The results of this study support an additional role of calprotectin in assessing inflammatory activity in patients with RA.

## Introduction

Rheumatoid arthritis (RA) is the most common chronic inflammatory joint disease, affecting approximately 1% of the world’s population [1]. The course of RA can be very heterogeneous, leading to variable outcomes, spanning from mild synovitis to severe joint damage with irreversible structural changes and functional impairment. RA appears to be a heterogeneous disease with different pathogenic mechanisms involved, in which the infiltration and activation of inflammatory cells and the production of a wide range of inflammatory mediators play significant roles [2, 3]. Several investigators have suggested calprotectin as a promising marker of disease activity in several inflammatory diseases in which myeloid cells play a crucial role [4, 5]. Calprotectin (also known as the S100 proteins, S100A8/S100A9 [6], MRP8/MRP14 [4], calgranulin A and B [7], and L1 protein [8]) is a major leukocyte cytosolic protein released locally during inflammatory processes predominantly by activated leukocytes at the site of joint inflammation and thus directly reflects joint inflammatory activity rather than systemic inflammation [9]. Calprotectin has been identified as an important endogenous alarmin, one of the damage-associated molecular pattern (DAMP) molecules that acts as a ligand for the TLR4 receptor [10] and amplifies the inflammation cascade via NF-kB and p38 mitogen-activated protein kinase. High concentrations of this protein were found in the synovial fluid of active arthritic joints [11]. Several authors have reported increased calprotectin serum levels in RA patients, its association with disease activity [5, 12], its dynamic decrease after initiation of effective treatment [13] and its role in predicting response to treatment [14, 15]. In addition, calprotectin has been shown to correlate with ultrasound-determined synovitis [16], which is considered to be a more sensitive tool for evaluating RA activity [17–19]. It has even been demonstrated that serum calprotectin may identify patients in clinical remission with subclinical disease activity detected by ultrasonography [20]. Recently, we have reported that calprotectin might be superior to C-reactive protein (CRP) in predicting ultrasound synovitis and suggested that calprotectin might be a valuable marker of RA disease activity [21]. However, the number of subjects studied previously was rather small, and this suggestion required further validation in larger cohorts.

The aims of this study were as follows: (1) to validate a strong association of calprotectin with traditionally used markers of disease activity, (2) to validate a strong association of calprotectin with ultrasound-determined synovitis in a larger cohort, and (3) to determine the predictive value of calprotectin and CRP for clinical disease activity (CDAI index) and power Doppler (PD US) synovitis.

## Methods

### Patients

A total of one hundred sixty patients fulfilling the ACR/EULAR 2010 classification criteria for RA were recruited from the outpatient department of rheumatology at the Institute of Rheumatology in Prague, from January 2012 to November 2015, and were consecutively enrolled in this study [22]. The study was performed according to the guidelines of the Declaration of Helsinki and was approved by the local ethics committee of the Institute of Rheumatology in Prague. All included patients gave their informed written consent before entry into the study. Joint assessment was performed in all patients by an experienced study nurse. Disease activity was evaluated by the Disease Activity Score (DAS28 score) [23] using swollen joint count (SJC) and tender joint count (TJC), erythrocyte sedimentation rate (ESR) or C-reactive protein (CRP) and global assessment of the patient using the visual analogue scale (VAS), Simplified Disease Activity Index (SDAI) [24] and Clinical Disease Activity Index (CDAI) [25].

### Laboratory assessment

Blood samples were obtained on the same day that the ultrasound examination was performed. The serum samples were centrifuged and stored at –80°C until analysis. Calprotectin was measured by a commercially available enzyme-linked immunosorbent assay (ELISA) according to the manufacturer’s instructional protocol (Bühlmann Laboratories AG, Schőnenbuch, Switzerland). The inter-assay and intra-assay reliability of the S100A8/9 assays were 5.8% and 4.3%, respectively, and the detection limit was 0.4 μg/mL. ESR with normal level < 20 mm/h was measured on a BD-15^™^ instrument (BD, New Jersey, USA). The CRP level (normal level < 5 mg/L) was measured using turbidimetry (Beckman Coulter, California, USA). Anti-cyclic citrullinated peptide (anti-CCP) antibodies were analysed using standard ELISA kits (Test Line s.r.o., Brno, Czech Republic).

### Ultrasound imaging

For the ultrasound examination, the German US7 score was applied. The patients were examined in the following seven joint areas: wrist and second and third metacarpophalangeal (MCP), second and third proximal interphalangeal (PIP) and second and fifth metatarsophalangeal joints of the clinically more affected hand and foot [26]. We used a modification of the original German US7 scoring system. In contrast to the original US7 that examines synovitis of the MCP and PIP joints in the Grey Scale (GS) only from the palmar view, we assessed synovitis in these areas from both the palmar and dorsal views. Moreover, we assessed tenosynovitis/paratenonitis only from the palmar aspect. Synovitis in the GS and PD as well as tenosynovitis in PD were scored semiquantitatively (0–3) as described earlier [21]. Tenosynovitis in the GS was evaluated as absent (0) or present (1). Overall GS and PD signal scores were calculated as the sum of GS synovitis, PD synovitis, GS tenosynovitis and PD tenosynovitis. The ultrasound examinations were carried out using high-sensitivy ultrasound equipment Esaote Mylab 60 instrument (Esaote S.p.A., Genova, Italy), with a frequency range from 12 to 18 MHz. The ultrasonographers were unaware of each patient’s clinical examination and laboratory findings. Our inter- and intra-observer reliability has recently been published with moderate to very good results [21].

### Statistical analysis

The data are described as the mean and standard deviation (SD), unless stated otherwise. A Kolmogorov-Smirnov test of normality was performed for all variables. The bivariate relationship between the variables was assessed using Spearman’s correlation coefficient. In order to analyse the differences between groups of interest, the independent samples *t*-test (or Mann—Whitney test as a nonparametric alternative) and ANOVA (or Kruskal—Wallis test as a nonparametric alternative) were used, followed by post-hoc tests with Bonferroni correction. In addition, linear regression analysis was performed for all examinations with the CDAI or PD sum score as the dependent variable and with SJC, age, sex, disease duration and RF-IgA titres as well as each of the biomarkers (one at a time) as the independent variables. For all statistical evaluations, *P* values below 0.05 were considered to be statistically significant. Statistical analyses were performed using SPSS version 23 statistical software (SPSS, Inc., Chicago, IL, USA).

## Results

### Baseline patient characteristics

The clinical, laboratory and ultrasound characteristics of the patients are shown in Table 1. The study population was predominantly female (80%) with a mean age of 55 ± 13.5 years (with range from 20.3 to 90 years). The mean (SD) disease duration was 6.4±6.3 years from initial symptoms. At the time of examination, 28 patients had high active disease (DAS28 > 5.1), 50 patients had moderate disease activity (3.2 < DAS28 ≤ 5.1), 23 patients had low disease activity (2.6 ≤ DAS28 < 3.2) and 59 patients were in remission (DAS28 < 2.6). Rheumatoid factor (RF) and anti-CCP positivity were found in 57.5% (92/160) and 56.3% (90/160) of RA patients, respectively.

**Table 1:** Baseline characteristics of the patients with rheumatoid arthritis.

Characteristics	RA patients (n = 160)		
Female (%)	128		80%
Age (years)	55	±	13.5
Disease duration (years)	6.4	±	6.3
Calprotectin, μg/mL	3.3	±	4
CRP, mg/L	9	±	19
ESR, mm/1^st^ hour	19.4	±	17
DAS28-ESR score	3.4	±	1.5
DAS28-CRP score	3.2	±	1.4
SDAI score	13	±	11.8
CDAI score	12.1	±	10.7
	4.9	±	4.6
PD syn score	2.7	±	3.6
GS ten score	0.4	±	0.8
PD ten score	0.4	±	1.2
RF positivity, n (%)	92/160		57.5%
Anti-CCP positivity, n (%)	90/160		56.3%
Glucocorticoids, n (%)	61/160		38.1%
csDMARDs, n (%)	140/160		87.5%
bDMARDs, n (%)	4/160		2.5%

The values are the mean ± SD (range), unless stated otherwise.

Anti-CCP, anticyclic citrullinated peptide antibody; bDMARDs, biologic disease-modifying antirheumatic drugs; CDAI, Clinical Disease Activity Index; CRP, C-reactive protein; csDMARDs, conventional synthetic disease-modifying antirheumatic drugs; DAS28-CRP, Disease Activity Score for 28 joints with C-reactive protein; DAS28-ESR, Disease Activity Score for 28 joints with erythrocyte sedimentation rate; ESR, erythrocyte sedimentation rate; F, female; GS syn score, Grey Scale synovitis score; GS ten score, Grey Scale tenosynovitis score; PD syn score, Power Doppler synovitis score; PD ten score, Power Doppler tenosynovitis score; RA, rheumatoid arthritis; RF, rheumatoid factor; SDAI, Simplified Disease Activity Index; SJC, swollen joint count; and TJC, tender joint count.

In our cohort, 27 patients were current smokers, 43 were past smokers and 76 never smoked. For 14 patients, data regarding their smoking status were missing.

### Relationship between traditional measures of disease activity and ultrasound scores

**Fig 1:**
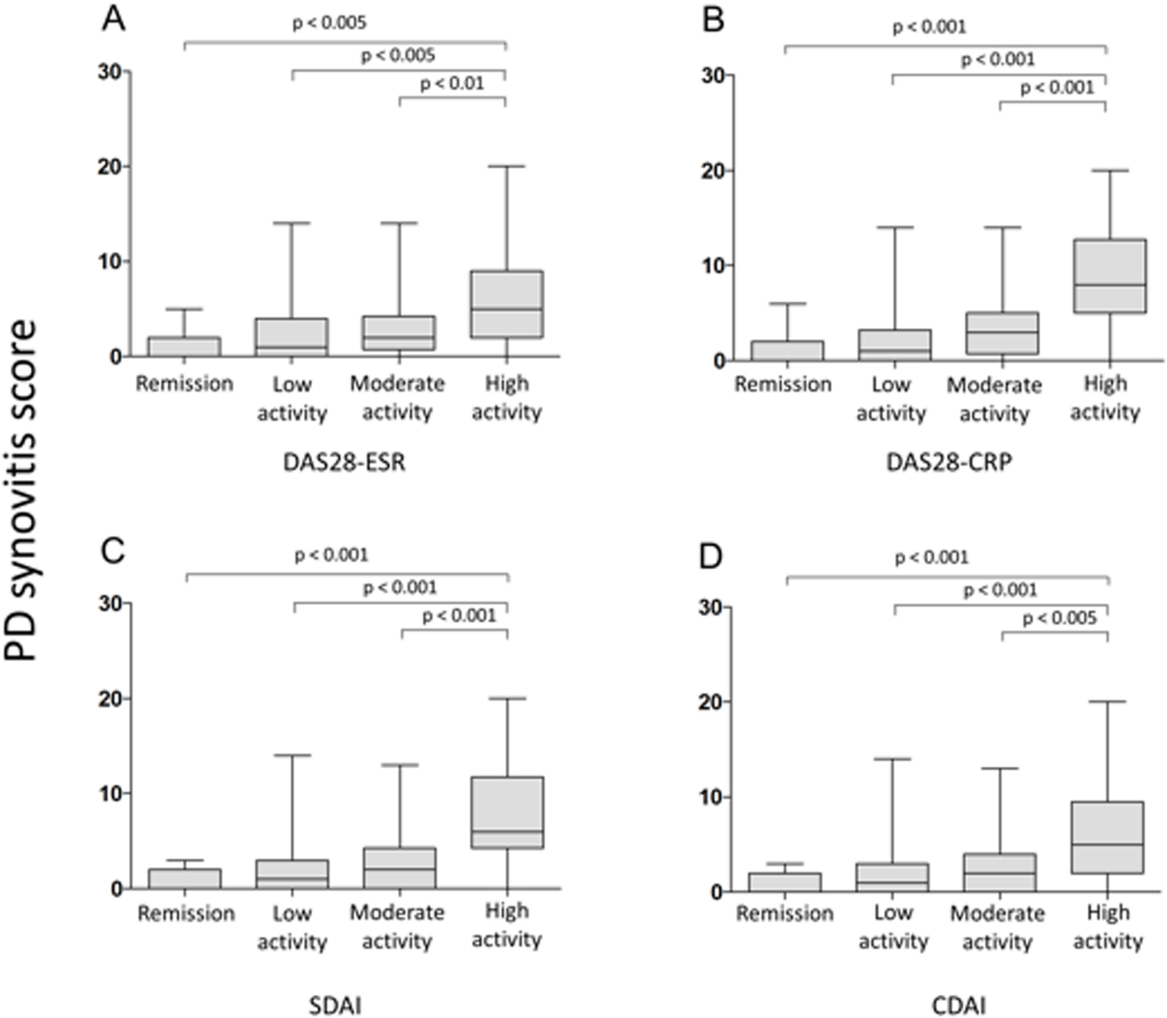
Box plots showing the associations between Power Doppler synovitis score and disease activity according to A) DAS28-ESR, B) DAS28-CRP, C) SDAI, and D) CDAI.

In the cross-sectional analyses, we found a moderate to strong correlation of ultrasound measures such as the GS and PD synovitis scores with TJC, SJC, DAS28-ESR, DAS28-CRP, SDAI, CDAI, CRP and ESR (Fig 1) (Table 2). Furthermore, we found an association between the GS and PD tenosynovitis scores with TJC, SJC, DAS28-CRP, CRP, and SDAI and between the GS tenosynovitis score and CDAI (Table 2).

**Table 2:** Spearman‘s rank correlation coefficients between serum calprotectin levels and clinical, laboratory and ultrasound parameters.

Parameter	CRP	ESR	DAS28-ESR	DAS28-CRP	SDAI	CDAI	GS syn	PD syn
Calprotectin	0.556**	0.380**	0.321**	0.346**	0.305**	0.279**	0.379**	0.419**
CRP		0.624**	0.418**	0.465**	0.363**	0.309**	0.373**	0.386**
ESR			0.611**	0.420**	0.402**	0.355**	0.291**	0.272**
DAS28-ESR				0.951**	0.942**	0,928	0.520**	0.484**
DAS28-CRP					0.968**	0.960**	0.549**	0.515**
SDAI						0.996**	0.571**	0.499**
CDAI							0.548**	0.490**
GS syn								0.661**

CDAI, Clinical Disease Activity Index; CRP, C-reactive protein; DAS28-CRP, Disease Activity Score for 28 joints with CRP; DAS28-ESR, Disease Activity Score for 28 joints with ESR; ESR, erythrocyte sedimentation rate; GS syn, Grey Scale synovitis score; GS ten, Grey Scale tenosynovitis score; SDAI, Simplified Disease Activity Index; PD syn, Power Doppler synovitis score; and PD ten, Power Doppler tenosynovitis score.

*Correlation is significant at the p<0.05 level

**Correlation is significant at the p<0.01 level

### Relationship between calprotectin and clinical, laboratory and ultrasound parameters of disease activity

**Fig 2:**
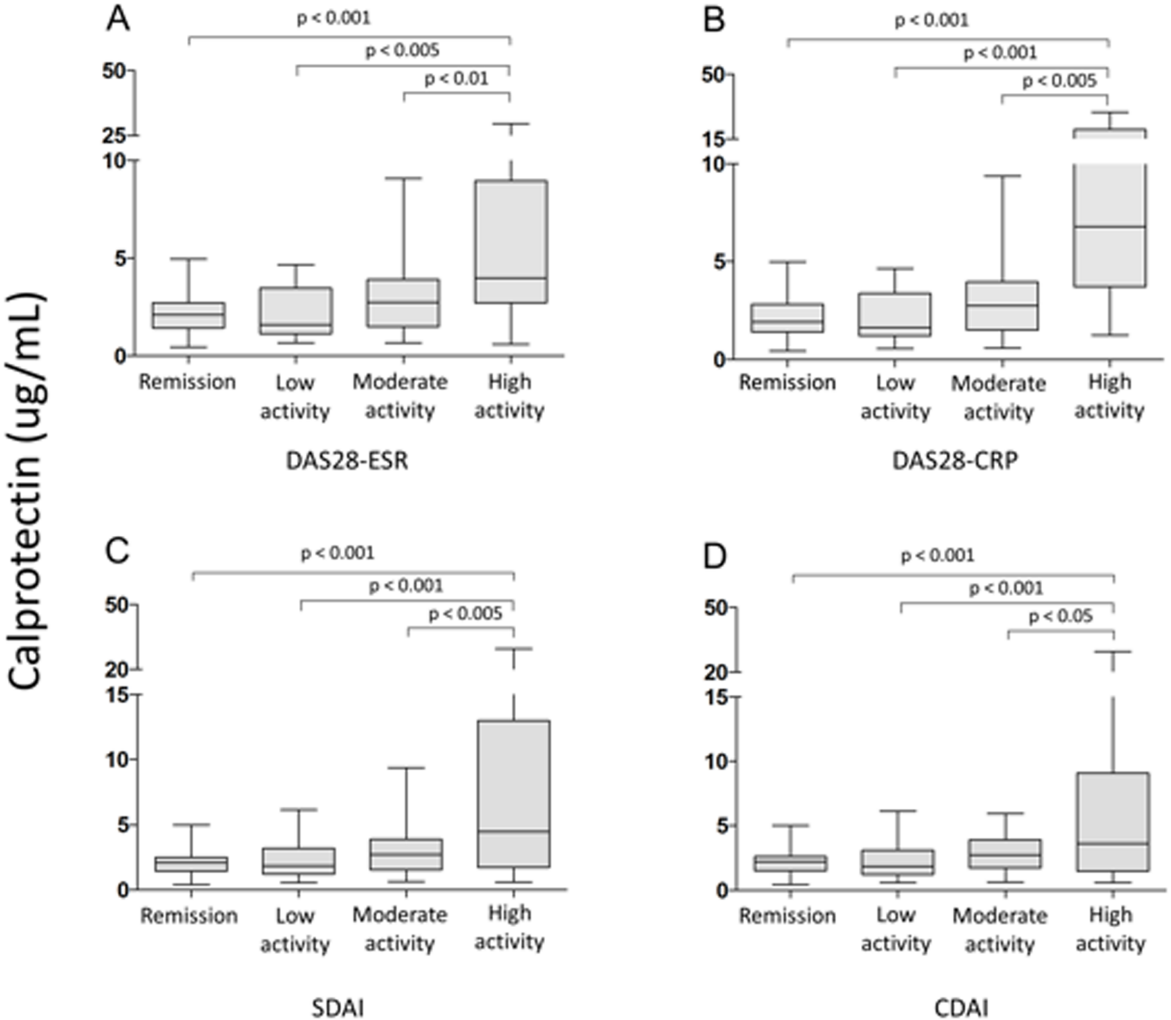
Box plots showing the associations between calprotectin serum levels and disease activity according to A) DAS28-ESR, B) DAS28-CRP, C) SDAI, and D) CDAI.

Serum calprotectin levels significantly correlated with TJC, SJC, DAS28-ESR, DAS28-CRP, SDAI, CDAI, ESR and, in particular, CRP levels (Fig 2) (Table 2). Calprotectin was significantly associated with the GS and, more importantly, with the PD synovitis score (Table 2). There was no association between serum calprotectin levels and either GS or PD tenosynovitis scores.

Serum calprotectin levels were higher in RF-positive than in RF-negative patients (mean 3.66 ± 4.89 μg/mL vs. 2.83 ± 2.24 μg/mL, respectively); however, these differences were not statistically significant. There was no difference in calprotectin levels between ACPA positive and ACPA negative patients (mean 3.35 ± 4.1 vs. 3.3 ± 2.27 μg/mL, respectively). Calprotectin serum levels were associated with IgA-RF (r = 0.184, p<0.05), but not with anti-CCP, RF-IgM nor RF-IgG levels in this cohort.

### Predictive value of calprotectin and C-reactive protein for CDAI and PD US activity

Different models of regression analysis were computed. One model used CDAI as a dependent variable, while the second model used the PD US synovitis score. In each model, serum biomarkers were used separately (one at a time) to avoid potential collinearity. Calprotectin (β = 0.176, p = 0.007) along with SJC (β = 0.731, p<0.001) (R^2^ = 0.677, adjusted R^2^ = 0.661), but not CRP (β = 0.092, p = 0.182), contributed significantly to explain CDAI activity as the dependent variable in the linear regression analyses (S1 Table)

With the PD US synovitis activity as the dependent variable, both serum markers, calprotectin (β = 0.499, p<0.001) and CRP (β = 0.349, p<0.001), along with SJC (β = 0.374, p<0.001 in model with calprotectin; β = 0.390, p<0.001 in model with CRP) contributed significantly; however, calprotectin contributed the most among all the independent variables. Moreover, the regression model with calprotectin had a higher squared multiple correlation than the model with CRP (R^2^ = 0.490, adjusted R^2^ = 0.465; R^2^ = 0.414, adjusted R^2^ = 0.385, respectively) (S2 Table). For all the models used in this study, the increase in R^2^ for predicting CDAI or PD synovitis score was not significantly affected by adding sex, age, disease duration or RF titres into the regression model.

### Association between smoking and ACPA levels, calprotectin levels and clinical disease activity

In our cohort, past smokers (n = 43) together with current smokers (n = 27) had higher levels of ACPA antibodies than patients who never smoked (n = 76) (mean 715.37 ± 1299 vs. 555.2 ± 987 U/mL respectively); however, this difference was not statistically significant. Furthermore, ACPA levels were higher in the current smokers than in the current non-smokers (mean 967.2 ± 1439 vs. 558.5 ± 1064 U/mL, respectively).

Similarly, calprotectin levels (mean 3.5 ± 4.4 μg/mL vs. 2.9 ± 3.4 μg/mL, respectively) along with SJC, DAS28-ESR (mean 3.56 ± 1.42 vs. 3.3 ± 1.5, respectively), and DAS28-CRP (mean 3.25 ± 1.3 vs. 3.08 ± 1.37, respectively) were higher in past smokers and current smokers than in patients who never smoked, generally reflecting the fact that smokers have higher disease activity than non-smokers.

In contrast, CRP levels did not differ in patients according to their smoking status.

## Discussion

We have validated the significant association of serum calprotectin with clinical and laboratory markers of disease activity in a large cohort; and more importantly, with ultrasound synovitis, which is considered to be more precise in the evaluation of joint inflammation. Furthermore, we have shown that calprotectin is a better predictor of CDAI activity and PD US synovitis than CRP and might have an additional role compared with the traditional acute phase proteins.

Calprotectin is a promising serum marker of inflammatory activity in RA with potential superiority over CRP [5]. Calprotectin differs from other laboratory markers by its local production from activated synovial cells and release from inflamed synovium [9, 27, 28]. As a consequence, calprotectin directly reflects the amount of activated macrophages and the extent of inflammation. Moreover, calprotectin is a small molecule with a molecular weight of 36.5 kDa that may easily diffuse from inflamed joints into circulation, where it can be easily measured [29]. Calprotectin is relatively stable and can be measured without the need for cold storage, making it a feasible marker for wider use in clinical practice [14].

In the last two decades, calprotectin has been studied by several research groups, and the significant association between serum calprotectin and conventionally measured acute-phase reactants (CRP, ESR) [30, 31] as well as clinical variables of disease activity in patients with RA has been shown [5, 12]. It would be of great interest to clarify whether serum calprotectin might have an additional role in assessing RA activity or if it is an alternative marker to traditionally measured acute phase reactants. Some studies have demonstrated that calprotectin might be a more sensitive marker of inflammation than CRP. For example, we have demonstrated that a decrease in the serum calprotectin level rather than in the CRP level reflected improvement in SJC after successful treatment in patients with recent-onset RA [13]. Other studies have reported that calprotectin is a more reliable biomarker of disease activity than acute-phase reactants in RA patients receiving TNF inhibitors or tocilizumab [32, 33].

In the present study, we have demonstrated a statistically significant relationship among the studied biomarkers and between serum markers and different composite clinical indices (DAS28, SDAI and CDAI). As expected, both calprotectin and CRP serum levels correlated with all composite indices. However, CRP forms part of the DAS-CRP and SDAI constructs, and therefore, an association of CRP with these composite indices should be assessed with regard to this. Consequently, to elucidate which serum marker reflects better clinical activity, we used multiple regression analysis to predict clinical disease activity according to the CDAI index, which does not contain any serum markers. We found that calprotectin, but not CRP, was an independent and significant predictor of CDAI activity.

Furthermore, to ensure accurate assessments of joint inflammation, we provided ultrasound measurements of disease activity, which is widely accepted as a more sensitive tool for disease activity assessment than clinical examination itself. To date, there are limited data on the association between circulating calprotectin, CRP and ultrasound-verified synovitis. A significant correlation between calprotectin and ultrasound-determined synovial inflammation in RA patients was repeatedly demonstrated by Hammer. However, in both studies plasma calprotectin concentrations were analysed [16, 34]. We have previously reported even stronger correlations between circulating serum calprotectin and ultrasound synovitis and demonstrated that calprotectin is a better predictor of ultrasound synovitis than conventionally used CRP [21]. Another study by Inciarte-Mundo et al. also reported a close association between serum calprotectin and ultrasound parameters and showed that calprotectin may help identify power Doppler activity in RA and psoriatic arthritis patients in clinical remission or low disease activity [35]. Very recently, we have demonstrated that serum calprotectin discriminates subclinical disease activity from ultrasound-defined remission in patients with RA in clinical remission [20]. However, most of previous studies on the associations between serum calprotectin and ultrasound-determined synovitis examined rather small cohorts of patients and need further validation.

In this large cohort study, we validated a significant correlation between calprotectin and GS synovitis, and more importantly, with PD synovitis reflecting active joint inflammation. Notably, serum calprotectin correlated better with both ultrasound variables than CRP. Moreover, multiple regression analysis showed that calprotectin is a better predictor of PD US synovitis than CRP.

The limitations of our study include its cross-sectional character and the participation of more ultrasonography investigators; however, as we already published, our inter- and intra-reader reliability was moderate to very good.

## Conclusions

The results of this study contribute to the growing evidence that calprotectin has an additional role compared to conventionally used serum markers in assessing RA activity.

## Supporting information

S1 Table Multiple regression analyses predicting Clinical Disease Activity Index (CDAI) by calprotectin (A) and C-reactive protein (B) levels. (XLSX)

S2 Table Multiple regression analyses predicting Power Doppler synovitis (PD US) score by calprotectin (A) and C-reactive protein (B) levels. (XLSX)
